# Alcohol Consumption and Viral Load Are Synergistically Associated with CIN1

**DOI:** 10.1371/journal.pone.0072142

**Published:** 2013-08-19

**Authors:** Kyung-Jin Min, Jae-Kwan Lee, Sanghoon Lee, Mi Kyung Kim

**Affiliations:** 1 Department of Obstetrics and Gynecology, Inha University Hospital, Incheon, Korea; 2 Department of Obstetrics and Gynecology, Korea University College of Medicine, Seoul, Korea; 3 Carcinogenesis Branch, Division of Cancer Epidemiology and Management, National Cancer Center, Goyang, Korea; IPO, Inst Port Oncology, Portugal

## Abstract

**Purpose:**

We investigated the association between alcohol consumption and risk of cervical intraepithelial neoplasia (CIN) and cervical cancer, and determined whether these associations were modified by human papillomavirus (HPV) viral load in high-risk HPV-positive women participating in the Korean HPV cohort study (KHPV).

**Methods:**

Among the women recruited in the KHPV (*n* = 1,243) from March 2006 to December 2009, we analyzed normal cytology (*n = *581) as control group, CIN1 (*n = *299), CIN2/3 (*n* = 161), or cervical cancer (*n* = 202). Multinomial logistic analysis was performed to estimate multivariate-adjusted odds ratios (OR).

**Results:**

Alcohol drinkers had an increased risk of CIN1 (OR = 2.18, 95% CI 1.22–3.89) compared with non-drinkers after adjusting for potential confounders. Subjects with more frequent alcohol consumption had a higher risk of CIN1 (*p* for linear trend <0.0001). Higher ethanol consumption was associated with an increased risk of CIN1 (*p* for linear trend = 0.0001). We also observed a synergistic effect between HPV viral load and alcohol consumption: drinkers with a high HPV viral load (≥100 RLU/PC) were associated with a significantly increased risk of CIN1 (OR = 19.1; 95% CI, 6.60–55.3, interaction *p*<0.001). There were no associations between alcohol drinking and CIN2/3 or cervical cancer.

**Conclusions:**

HPV viral load and alcohol was associated with the risk of CIN1 among high-risk HPV-positive women. This is the first demonstration that alcohol is an independent and combined risk factor of CIN1.

## Introduction

Although human papillomavirus (HPV) infection is one of the major causes of cervical intraepithelial neoplasia (CIN) and cervical cancer, most HPV infections are asymptomatic and transient and therefore other cofactors in conjunction with HPV infection are necessary for progression to malignant disease [1]. The American Cancer Society reports the following risk factors for cervical cancer besides HPV: smoking, HIV infection, chlamydia infection, stress and stress-related disorders, dietary factors, hormonal contraception, multiple pregnancies, exposure to the hormonal drug diethylstilbestrol, and family history of cervical cancer. However, the potential role of alcohol consumption in the etiology of cervical cancer has not been extensively studied [2].

The International Agency for Research on Cancer of the World Health Organization has classified alcohol as a Group 1 carcinogen [3]. High intake of alcohol increases the risk of cancer in multiple organs, including upper gastrointestinal tract, lung, liver, large bowel, and breast [4]–[7]. However, the relationship between alcohol and cancer remains controversial depending on the site of the malignancy.

Limited evidence exists on the potential role of alcohol drinking in the carcinogenesis of cervical cancer and its precancerous lesions [2]. There is no prior study on the interaction between HPV and alcohol drinking with regard to development of CIN and progression to malignancy. The purpose of this Korean HPV cohort study (KHPV) is to investigate the associations between alcohol consumption and the risk of CIN and cervical cancer, and to assess the combined effect of alcohol and HPV infection in high-risk HPV-positive women.

## Materials and Methods

### Study Population

The KHPV has been conducted with approval from the Korean National Cancer Center institutional review board (NCCNCS-06-062). Subject, aged 18–65, participated in a HPV cohort study from March 2006 up to present and all participants provided the written informed consent as each institutional review boards’ approval. The study was approved by the ethics committees of the Korean National Cancer Center and Korea University Guro Hospital. Participants were randomly selected from the gynecologic oncology clinics of university hospitals in Korea. Women were eligible to participate if they were currently sexually active or seeking birth control, were not currently pregnant, had an intact uterus, had no current referral for hysterectomy, and had no history of treatment for cervical intraepithelial neoplasia within the previous 18 months. Exclusion criteria included a history of gynecological cancers such as cervical, ovarian, or endometrial cancer; insufficient data on the questionnaire; inadequate blood for evaluation; a chronic disease such as liver cirrhosis, renal failure, or cardiovascular disease; drug dependency; or psychological problems. We analyzed baseline data of the total cohort as described in our previous study [8], [9].

**Figure 1:**
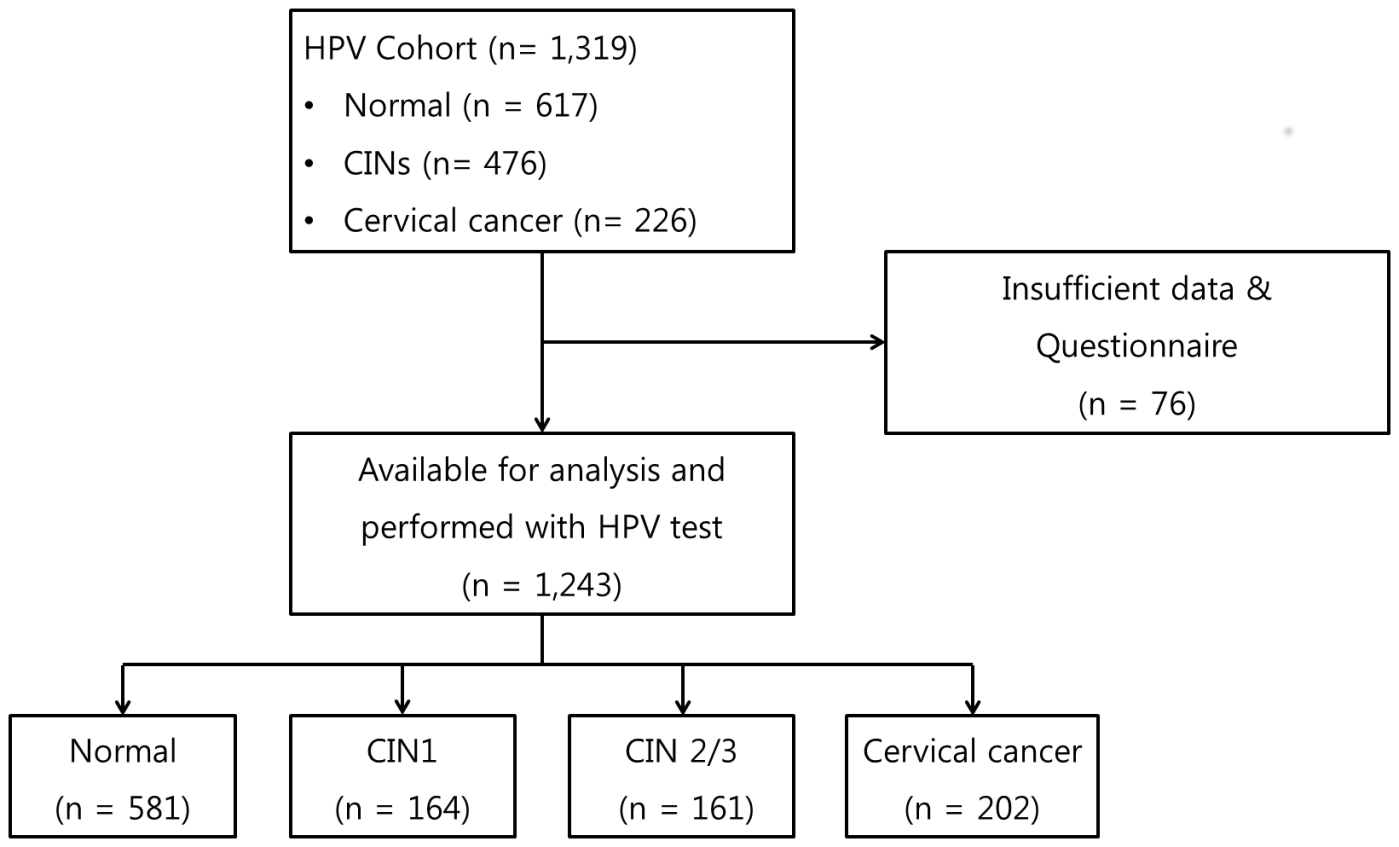
Study subjects enrolled in the present analysis.

Among 1,374 enrolled women, 1,319 met the inclusion criteria. Sufficient questionnaire data were not available for 76 women. HPV testing with Hybrid capture 2 (HC 2, Digene Diagnostics, Gaithersburg) was performed in 1,243 women and a total of 674 (54.2%) women were positive with oncogenic HPV (HPVs 16, 18, 31, 33, 35, 39, 45, 51, 52, 56, 58, 59, and 68). Total participants were included in the present analysis (Fig. 1).

### Pap Smear Cytology, HPV DNA Test, and Histologic Examination

Gynecologic examinations, HPV DNA tests, liquid-based cervical cytology, and measurement of viral load as relative light units/positive control (RUL/PC) were performed at the first enrollment and each subsequent visit. The cytologic grade for Pap smear reports was based on the Bethesda classification system [10]. Colposcopic examinations and histologic verifications were performed at baseline and during follow-up visits in all women with abnormal cervical cytology.

Normal cytological diagnosis at entry formed the control group (*n* = 581), and others were categorized into CIN1 (*n* = 299), CIN2/3 (*n* = 161), and cancer groups (*n* = 202) regardless of the results of HPV test. For CIN, the effect of alcohol consumption was examined for CIN1 and CIN2/3 subgroups respectively [8], [11]–[13]. HPV viral load was categorized into three groups: high (≥100 RLU/PC), medium (10–100 RLU/PC), and low (<10 RLU/PC) [14]–[17].

### Data Collection

At study entry, participants responded to the risk factor questionnaire that assessed a wide range of information on demographic, socioeconomic, and other factors related to HPV infection and cervical neoplasia. The health habits questionnaire on alcohol intake included drinking status (current, former, never), frequency of consumption of five alcoholic beverages (beer, wine, hard liquor, soju, and makgeolli), age at which the subject started to drink alcohol, and duration of the drinking habit. If participants had stopped drinking, information on the duration of alcohol abstinence and the frequency of previous alcohol consumption was obtained. Beverage-specific alcohol consumption was calculated in g/day based on alcohol content of the five beverages, the frequency of drinking, and the amount consumed. We calculated the total alcohol consumption (g/d) by summing the beverage-specific amount consumed, and the total liquor consumption (g/d) by summing the consumed amount of different beverage types.

### Statistical Analyses

The χ^2^ test and ANOVA were used for analysis of differences in the distribution of categorical and continuous variables, respectively. Multinomial logistic regression models were used to estimate odds ratios (ORs) and corresponding 95% confidence intervals (CIs). Linear trends were calculated using the median values for each risk factor as a continuous variable. Multinomial logistic regression model was performed to evaluate the association between alcohol and the risk of CINs and cervical cancer after adjustment for age (10-year age groups), smoking habit (never vs. ever), oral contraceptive use (never vs. ever), number of children (0, 1, 2, ≥3), physical activity (METS: <170, 170–218, and ≥218), and energy intake from food (kcal/day). Interaction tests were performed using multiplicative interaction terms of the ordinal score for viral load of HPV and alcohol drinking in the model. Ethanol intake level was categorized into two groups based on the distribution among the control participants, and risk estimates were calculated with the non-drinker group as the reference category. Joint effects of HPV and alcohol were estimated using relative excess risk due to interaction (RERI), attributable risk proportion due to interaction (AP), and synergistic index (SI) and their respective 95% CI [18], [19]. RERI is an estimate of excess risk that is directly attributable to the interaction between two exposures. AP is the proportion of risk that is attributable to the interaction between the two exposure variables. SI is a ratio that estimates whether a synergistic (SI>1) or antagonistic (SI<1) interaction exists between two exposures. A multiplicative interaction is suggested by the following scores: RERI >1.5, AP>0.25, and SI>1.5 [19]. All analyses were conducted using SAS (SAS Institute, Cary, NC, USA) and Intercooled Stata/SE (StataCorp. 2001, Stata Corporation, Stata Statistical Software: Release 10.0. College Station, TX, USA).

## Results

The sociodemographic, reproductive, anthropometric, and lifestyle characteristics of the study subjects were described in Table 1. There were statistically significant differences in age, body mass index, percentage of alcohol consumption, smoking status and amount, and physical activity except oral contraceptive use. Patients with cervical dysplasia (CIN1 and CIN2/3) were younger than control group and cervical cancer group. Women in the cervical dysplasia groups (7.50 g for CIN1, 6.31 g for CIN2/3) consumed a significantly higher amount of alcohol per day than the control (3.53 g) and cancer groups (3.88 g) (*p*-value <0.0001).

**Table 1:** Sociodemographic, reproductive, anthropometric, and lifestyle characteristics of study subjects.

Characteristic	Control	CIN1	CIN2/3	Cervical cancer	*p* *
	*n* = 581	*n* = 299	*n* = 161	*n* = 202	
Age (y), mean±SD	45.2±10.3	39.6±11.1	40.6±10.5	50.8±11.7	<0.0001
Body mass index (kg/m^2^), mean±SD	21.2±1.33	21.6±1.37	21.5±1.42	20.7±1.50	<0.0001
Alcohol consumption (% ever)	53	75.9	65.2	50	<0.0001
Total alcohol (g/day), mean ± SD	3.53±8.63^a^	7.50±16.4	6.31±13.6^b^	3.88±8.45^a^	<0.0001
From Soju (g/day), mean ± SD	1.74±4.86	3.80±8.89^b^	2.93±6.69	2.50±6.18	0.0001
From Beer (g/day), mean ± SD	1.14±3.17^a^	2.81±9.01^b^	2.59±6.77^b^	1.14±3.40^a^	<0.0001
From Wine (g/day), mean ± SD	0.17±0.65	0.28±0.99	0.17±0.85	0.12±0.92	0.12
From Liquor (g/day), mean ± SD	0.42±5.20	0.40±2.73	0.51±2.81	0.02±0.16	0.6
From Makgeolli (g/day), mean ± SD	0.07±0.38^a^	0.20±1.09^b^	0.11±0.54^b^	0.09±0.56^a^	0.04
Smoking status (% ever)	10.7	17.7	18	12.9	0.01
Pack-years of smoking (%)					0.04
Never	89.3	82.8	82	87.1	
0–20	8.4	14.8	13.7	9.4	
20–40	2.3	2.4	4.3	3.5	
No. of childbirths, mean±SD	2.03±0.92^a^	1.98±0.91^b^	2.04±0.87^b^	2.59±1.32^b^	<0.0001
Oral contraceptive use (% ever)	13.9	16.8	21.7	17.9	0.09
Education (% post college)	39	43.9	26.9	10.9	<0.0001
Age at menarche (y), mean±SD	14.5±1.86^a^	14.3±1.81^b^	14.5±1.88^a^	15.3±1.87^b^	<0.0001
Physical activity (METs/wk)*, mean±SD	61.2±44.1	57.1±53.1	51.6±34.8	70.8±90.9	0.0061
Total energy intake (kcal/day), mean±SD	1886±558^a^	1999±564^b^	1948±554^a^	1848±591^a^	0.009
Energy from alcoholic beverages (kcal/day)	24.7±60.4^a^	52.5±115^b^	44.2±95.4^b^	27.1±59.2^a^	<0.0001
Energy from food (kcal/day)	1861±553^a^	1946±566^b^	1904±531^a^	1821±583^a^	0.06

CIN cervical intraepithelial neoplasia, HPV human papillomavirus, MET metabolic equivalent tasks,

* ANOVA (duncan’s) for continuous variables and χ2 for categorical variables. P values are from Kruskal-Wallis test.

a,b means with different superscripts are significantly different from each other.

The multivariable-adjusted ORs for CIN1, CIN 2/3, and cervical cancer with respect to alcohol drinking were shown in Table 2. Alcohol drinking was significantly associated with the risk of CIN1 only. Neither CIN 2/3 nor cervical cancer showed an association with alcohol drinking. Compared with non-drinkers, drinkers with a high frequency and large amount of alcohol consumption had an increased risk of CIN1 (OR = 2.18, 95% CI: 1.22–3.89) after adjusting for potential confounders.

**Table 2:** Association between alcohol drinking and cervical dysplasia and cancer.

	Normal (*n* = 581)	CIN1 (*n* = 299)		CIN 2,3 (*n* = 161)		Cervical cancer (*n* = 202)	
	n (%)	n (%)	OR (95% CI)*	n (%)	OR (95% CI)*	n (%)	OR (95% CI)*
Drinking behavior							
Non-drinker	293(50)	80(26)	1 (ref.)	68(42)	1 (ref.)	123(61)	1 (ref.)
Current/Former Drinker	288(50)	219(74)	2.18(1.22–3.89)	93(58)	1.25(0.84–1.84)	79(39)	1.05(0.74–1.49)
Frequency of alcohol consumption							
Non-drinker	293(50)	80(26)	1 (ref.)	68(42)	1 (ref.)	123(61)	1 (ref.)
<1/month	70(12)	40(13)	2.42(1.05–5.56)	20(12)	1.28(0.73–2.24)	14(7)	0.90(0.52–1.58)
1–3/month	114(20)	88(30)	2.07(1.04–4.09)	29(18)	1.02(0.62–1.71)	29(14)	0.97(0.61–1.54)
≥1/week	104(18)	91(31)	2.27(1.09–4.74)	44(28)	1.48(0.90–2.41)	36(18)	1.33(0.84–2.12)
*p* for linear trend			<0.0001		0.19		0.37
Ethanol (g/day)							
Non-drinker	293(50)	80(26)	1 (ref.)	68(42)	1 (ref.)	123(61)	1 (ref.)
<6.34	187(33)	130(43)	2.03(1.43–2.88)	51(32)	0.96(0.63–1.48)	43(21)	0.66(0.43–0.99)
≥6.34	101(17)	89(31)	2.24(1.48–3.38)	42(26)	1.24(0.76–2.03)	36(18)	1.07(0.66–1.74)
*p* for linear trend			0.0001		0.23		0.78
Ethanol from Beer (g/day)							
Non-drinker	293(55)	80(28)	1 (ref.)	68(51)	1 (ref.)	123(71)	1 (ref.)
Drinker	189(45)	158(72)	2.15(1.51–3.08)	65(49)	1.03(0.67–1.58)	51(29)	0.82(0.54–1.24)
Ethanol from Beer (g/day)							
Non-drinker	293(61)	80(34)	1 (ref.)	68(51)	1 (ref.)	123(71)	1 (ref.)
<6.36	146(30)	107(45)	1.95(1.33–2.84)	39(29)	0.85(0.53–1.36)	34(20)	0.69(0.43–1.09)
≥6.36	43(9)	51(21)	2.95(1.76–4.96)	26(20)	1.71(0.93–3.16)	17(9)	1.35(0.71–2.60)
*p* for linear trend			<0.0001		0.26		0.88
Ethanol from Soju (g/day)							
Non-drinker	293(63)	80(36)	1 (ref.)	68(54)	1 (ref.)	123(65)	1 (ref.)
Drinker	175(37)	142(64)	2.26(1.58–3.23)	59(46)	1.04(0.67–1.60)	65(35)	1.04(0.71–1.53)
Ethanol from Soju (g/day)							
Non-drinker	293(63)	80(36)	1 (ref.)	68(54)	1 (ref.)	123(65)	1 (ref.)
<3.3	85(18)	63(28)	2.13(1.39–3.26)	17(13)	0.68(0.37–1.24)	24(13)	0.77(0.45–1.30)
≥3.3	90(19)	79(36)	2.40(1.57–3.67)	42(33)	1.40(0.85–2.31)	41(22)	1.33(0.83–2.12)
*p* for linear trend			<0.0001		0.27		0.42

* Multinomial logistic regression model. Adjusted for age (10-year age groups), smoking habit status (never vs. ever), oral contraceptive use (never vs. ever), number of child births (0, 1, 2, ≥3), physical activity (METS: <170, 170–218, and ≥218), energy intake from food (kcal/day) and HPV.

ORs for CIN1 associated with drinking frequency of <1/month, 1–3/month, and ≥4/month were 2.42 (95% CI: 1.05–5.56), 2.07 (95% CI: 1.04–4.09), and 2.27 (95% CI: 1.09–4.74), respectively (*p* for linear trend <0.0001). Compared with non-drinkers, daily alcohol consumption was associated with an increased risk of CIN1 (OR = 2.24, 95% CI: 1.48–3.38 for the high consumption group [6.34 g ethanol/day], and OR = 2.03, 95% CI: 1.43–2.88 for the low consumption group [<6.34 g of ethanol/day], *p* for linear trend = 0.0001).

Stratification according to the type of alcoholic beverage showed similar results with respect to the risk of CIN1. Compared with non-drinkers, moderately higher ORs were observed for each alcoholic beverage although the type of alcoholic beverage did not significantly influence associations (OR = 2.15, 95% CI 1.51–3.08 for beer and OR = 2.26, 95% CI 1.58–3.23 for Soju). No associations were found between the type, amount, or frequency of alcohol drinking and CIN2/3 or cervical cancer.

Table 3 shows the combined effects of alcohol drinking and HPV viral load on the risk of CIN1. Independent effects of a higher HPV viral load and alcohol consumption were identified: compared with non-drinkers with the lowest HPV viral load (<10 RLU/PC), the multivariate OR (95% CI) was 4.94 (1.63–15.0) for non-drinkers with the highest HPV viral load (≥100 RLU/PC) and 2.10 (0.78–5.68) for drinkers with the lowest HPV viral load. Moreover, HPV viral load and alcohol drinking had a synergistic effect: drinkers with the highest HPV viral load (≥100 RLU/PC) had a significantly increased risk of CIN1 (OR = 19.1; 95% CI, 6.60–55.3, *p* for linear trend <0.001). The interaction between alcohol drinking and HPV viral for risk of CIN1 was statistically significant (*p*<0.0001). RERI, AP, and SI scores were 15.7 (95% CI: −3.95–35.4), 0.71 (95% CI: 0.45–0.96), and 3.89 (95% CI: 1.43–10.6), respectively, indicating a multiplicative interaction between HPV viral load and alcohol drinking.

**Table 3:** Combined effects of alcohol drinking and HPV viral load on the risk of CIN1 in HPV-positive women.

		CIN1		
		HPV viral load (RLU/PC^3^)		
		<10	10–100	≥100
Drinking habit				
Non-drinker	N(control/case)	35/7	16/11	16/16
	Multivariate OR (95% CI)*	1 (ref.)	3.99(1.20–13.3)	4.94(1.63–15.0)
Current/Former drinker	N(control/case)	46/27	21/25	13/78
	Multivariate OR (95% CI)*	2.10(0.78–5.68)	4.50(1.57–12.9)	19.1(6.60–55.3)
Multivariate interaction *p* value*			<0.0001	
*p* for linear trend			<0.0001	
RERI and 95% CI^2^			15.7(−3.95–35.4)	
AP and 95% CI^2^			0.71(0.45–0.96)	
SI and 95% CI^2^			3.89(1.43–10.6)	
Ethanol (g/day)				
Non-drinker	N(control/case)	37/7	16/11	17/19
	Multivariate OR (95% CI)*	1 (ref.)	4.19(1.27–13.9)	5.65(1.91–16.7)
Low (<6.34)	N(control/case)	29/20	14/16	8/42
	Multivariate OR (95% CI)*	2.75(0.98–7.76)	4.73(1.52–14.7)	19.7(6.15–62.9)
High (≥6.34)	N(control/case)	15/7	7/9	4/33
	Multivariate OR (95% CI)*	1.49(0.41–5.39)	4.84(1.23–19.1)	22.0(5.41–89.3)
Multivariate interaction *p* value*			<0.0001	
*p* for linear trend			<0.0001	
RERI and 95% CI^2^			16.7(−16.1–49.5)	
AP and 95% CI^2^			0.72(0.33–1.11)	
SI and 95% CI^2^			4.03(0.88–18.4)	
Frequency of drink consumption				
Non-drinker	N(control/case)	35/7	16/11	16/16
	Multivariate OR (95% CI)*	1 (ref.)	3.98(1.19–13.3)	5.00(1.64–15.2)
<1 time/week	N(control/case)	29/18	13/13	9/50
	Multivariate OR (95% CI)*	2.36(0.83–6.77)	3.52(1.08–11.5)	19.0(6.05–59.7)
≥1 times/week	N(control/case)	15/8	8/12	4/27
	Multivariate OR (95% CI)*	1.81(0.51–6.38)	6.39(1.77–23.1)	20.0(4.89–81.6)
multivariate interaction *p* value*			<0.0001	
*p* for linear trend			<0.0001	
RERI and 95% CI^2^			17.0(−15.2–49.1)	
AP and 95% CI^2^			0.72(0.35–1.09)	
SI and 95% CI^2^			4.08(0.95–17.6)	

* Multivariate logistic regression model. Adjusted for age (10-year age groups), smoking habit status (never vs. ever), oral contraceptive use (never vs. ever), number of childbirths (0, 1, 2, ≥3), physical activity (METS: <170, 170–218, and ≥218), and energy intake from food (kcal/day).

2 SI: synergic index (OR11-1)/(OR01+OR10-2), in which OR11 is the odds ratio of the joint effect of two risk factors and OR01 and OR10 are odds ratio of each risk factor in the absence of the other.

3 RLU/PC: relative light.

We further evaluated the combined effects of HPV viral load, alcohol consumption level, and frequency of drinking. A synergistic association with the risk for CIN1 was detected among subjects with high alcohol intake (>6.34 g/day) and the highest HPV viral load (≥100 RLU/PC) (OR = 22.0; 95% CI, 5.41–89.3, interaction *p*<0.0001), compared with non-drinkers with the lowest HPV viral load (<10 RLU/PC). A multiplicative interaction between HPV viral load and alcohol consumption level was revealed: RERI, AP, and SI values were 16.7 (95% CI, −16.1–49.5), 0.72 (95% CI, 0.33–1.11), and 4.03 (95% CI, 0.88–18.4), respectively. Compared with non-drinkers with the lowest HPV viral load (<10 RLU/PC), drinkers who consumed alcohol more frequently than once a week and had a higher HPV viral load (≥100 RLU/PC) had a significantly increased risk of CIN1 (OR = 20.0; 95% CI, 4.89–81.6, interaction *p*<0.0001). There was a multiplicative interaction between HPV viral load and the frequency of alcohol consumption: RERI, AP, and SI values were 17.0 (95% CI, −15.2–49.1), 0.72 (95% CI, 0.35–1.09), and 4.08 (95% CI, 0.95–17.6), respectively. There were no interactions between HPV viral load and alcohol drinking with respect to the risk of CIN2/3 and cervical cancer.

## Discussion

In this study, we showed that alcohol consumption and high HPV DNA viral load were associated with the risk of CIN1, but not CIN 2/3 or cervical cancer, in oncogenic HPV-positive women. To our knowledge, this is the first study to show a multiplicative interaction between HPV viral load and alcohol consumption in cervical carcinogenesis.

Some previous studies have reported the association between alcohol intake and high grade CIN or cervical cancer [20]–[22]. These studies investigated the association between alcohol intake and risk of high-grade squamous intraepithelial lesions (SILs) or cervical cancer, not low-grade SILs or CIN1. Generally, there is a J-shaped association between alcohol consumption quantity and the risk of all-cause and cancer deaths [23]. Relative to light drinkers (0.001∼12.8 g/day), never drinkers and heavy drinkers had an increased risk for all-cause and cancer deaths. Although controversy remains over whether alcohol is a risk factor for cervical cancer [24], [25] and even though alcohol consumption level in this study is light which categorized in other study [22], our results suggest that alcohol may be a cofactor for the development of CIN1. Benefits of mild consumption of alcohol have been suggested for several health outcomes, but controversies and inconsistent findings remain up to date [26]. As reported in a recent meta-analysis to investigate relationship between alcohol drinking and risk of all cancer mortality, there was a J-shaped association in males but not in females [27]. In a large-scale prospective study of Korean men and women, light-to-moderate alcohol consumption was associated with a lower mortality risk on all cause and cancer mortality in men, but did not show favorable effects on that in women [28]. The level of alcohol consumption among Korean women is relatively lower than those of other population. Asian population showed frequently a slow-metabolizing acetaldehyde dehydrogenase variant [29]. Furthermore, there is a gender difference in pharmacokinetics of alcohol and comorbidities derived from alcohol [30]. Therefore, Korean women having genetic susceptibility to alcohol might be different from other population in relation to association with health outcomes.

According to a recent evaluation of the carcinogenic risk to humans by the International Agency for Research on Cancer Monographs [31], oncogenic HPVs show sufficient epidemiological evidence of cervical carcinogenicity. Most cofactors appear to act as determinants of persistence and progression to advanced precursors, but no risk factor for progression to invasion has been established with the probable exception of age. This study indicated that HPV viral load and alcohol consumption were positively associated with the development of CIN1. Furthermore, our data suggested that increased risk of CIN1 was associated with a strong interaction between HPV viral load and alcohol consumption. After adjusting for potential cofactors, we present evidence that alcohol consumption and high HPV DNA viral load are significantly associated with the risk of developing CIN1 in high-risk HPV-positive women with normal cytology.

Since CIN1 could be regarded as a surrogate for persistent HPV infection, it is necessary to understand the pathology of high-risk HPV infection and prevent the initiation of cervical dysplasia processes that lead to cervical cancer. We found a significant association between alcohol drinking and CIN1, but not CIN2/3 or cervical cancer. A possible explanation for this is that alcohol decreases the number and cytotoxic activity of natural killer (NK) cells that eliminate tumor and pathogen-infected cells through the innate immune system [32]. A reduced number of NK cells and inhibition of cytokine production (interleukin-18-induced interferon-γ) in NK cells were observed in HPV-infected cervical tissue [12], [33], [34]. Davidson et al. reported that the suppression of inflammation was observed in CIN1 only, and may be associated with active viral replication [35]. Another possible explanation is the genetic linkage. It is well known that there is an association between multicentric carcinogenesis events in the upper aerodigestive tract including the esophagus and head and neck region in East Asia [36]. This can have relation with genetic polymorphisms of alcohol metabolizing enzymes (ALDH2 and ADH1B) that increased locally acetaldehyde a known carcinogen [37]. These polymorphic variants are prevalent in Japan as well as in other Asian countries [38]. Moreover, alcohol consumption is thought to increase the estrogen level [39], [40], which increases HPV expression via up-regulation of the progesterone receptor and the response to growth factors, and to facilitate cell proliferation [41], [42]. In summary, the present study provides epidemiologic support for the effects of alcohol on the development of CIN1 only. Alcohol does not seem to be affected the progression to high-grade cervical dysplasia and cervical cancer because the impacts of an impaired immune system and other effects of alcohol may be reduced after HPV is integrated into the host genome.

In our study, smoking status was significantly higher in CINs than control group by χ2 analysis, not in multivariate logistic regression. Smoking is recognized as a risk factor for HPV infection. But, it is still controversial that smoking is a risk factor for CIN [43]–[45]. Moreover, most of the research reported on the association smoking and high-grade CIN mainly. The report of Matsumoto et al included CIN1, but did not divide CINs [46]. One of possibilities about this discrepancy is that the ratio of non-smoker was quite higher ranged from 82.0% to 89.8% in four different groups due to the cultural reason in our study.

Our study has some specific strengths: to our knowledge this is the first multi-institutional epidemiologic study to investigate the development of CIN1 in Korean women with high alcohol intake and HPV viral load, and the study was performed by a trained investigator instead of using self-reports to obtain the drinking habit data. However, a limitation of our study was the lack of information on sexual contact in the questionnaire due to personal and cultural reasons. Alcohol consumption is probably associated with sexual behavior [47] and thus with the acquisition of HPV or other sexually transmitted infections [48]. Our study may overlook the fact that women with multiple sexual partners have greater opportunities for HPV acquisition. Another limitation is recall bias because the level of alcohol consumption was based on patients’ statements even though well-trained interviewers and comprehensive structured questionnaires were used.

In conclusion, we suggest that alcohol should be regarded as one of the risk factors for development of early cervical dysplasia in women with high-risk HPV infection. We recommend that clinicians encourage high-risk HPV-positive women with normal cytology to abstain from alcohol consumption to prevent the initiation of cervical dysplasia. Further study is needed to understand the exact mechanism by which alcohol affects the development of CIN1, and how a high viral load and other risk factors participate in progression to cervical cancer. Furthermore, we expect to contribute to the discovery and development of targets for chemoprevention through a large-scale study on the pathogenesis of progression to CIN1 induced by alcohol and HPV viral load.

### Previous Presentation

This work was presented at the 102^nd^ annual meeting of American Association for Cancer Research in 2011.
